# Impact of the Introduction of the Electronic Health Insurance Card on the Use of Medical Services by Asylum Seekers in Germany

**DOI:** 10.3390/ijerph15050856

**Published:** 2018-04-25

**Authors:** Kevin Claassen, Pia Jäger

**Affiliations:** 1 Faculty of Social Sciences, Ruhr-University Bochum, Universitätsstr. 150, 44801 Bochum, Germany; kevin.claassen@rub.de; 2 Department of Psychiatry Psychotherapy and Preventative Medicine, Faculty of Medicine, LWL-University Hospital, Ruhr-University Bochum, Universitätsstr. 150, 44801 Bochum, Germany

**Keywords:** public health, asylum seeker, Electronic Health Insurance Card, refugee, Germany

## Abstract

**Objectives:** Asylum seekers in Germany represent a highly vulnerable group from a health perspective. Furthermore, their access to healthcare is restricted. While the introduction of the Electronic Health Insurance Card (EHIC) for asylum seekers instead of healthcare-vouchers is discussed controversially using politico-economic reasons, there is hardly any empirical evidence regarding its actual impact on the use of medical services. The aim of the study is to examine this impact on the use of medical services by asylum seekers as measured by their consultation rate of ambulant physicians (CR). **Study Design:** For this purpose, a standardized survey was conducted with 260 asylum seekers in different municipalities, some of which have introduced the EHIC for asylum seekers, while others have not. **Methods:** The period prevalence was compared between the groups “with EHIC” and “without EHIC” using a two-sided *t*-test. Multivariate analysis was done using a linear OLS regression model. **Results:** Asylum seekers in possession of the EHIC are significantly more likely to seek ambulant medical care than those receiving healthcare-vouchers. **Conclusions:** The results of this study suggest that having to ask for healthcare-vouchers at the social security office could be a relevant barrier for asylum seekers.

## 1. Introduction

Worldwide there are 65.3 million people fleeing from war, violence and persecution [1]. In 2015 and 2016, a total of 1,222,194 refugees submitted a request for asylum in Germany. The German Federal Office for Migration and Refugees made 978,459 decisions about applications. The average operation time was 5.4 months [2,3]. A total of 21.21% of the asylum seekers were allocated to North Rhine-Westphalia in 2016 [4].

Overall, there is a complex relationship between flight and health, wherein there are many mutually influential factors [5]. While morbidity and mortality of migrants, which are similar to those of the majority population, are discussed as “healthy migrant effects”, there are some peculiarities among asylum seekers [6]. Among other things, there are increased rates of infectious diseases such as tuberculosis, HIV or hepatitis, scabies or measles [7,8,9].

Of particular relevance is the occurrence of mental illnesses and psychosocial stress. First, both the flight itself and its causes are linked to mental stress and disturbance patterns that may include risk factors, and secondly, they are particularly difficult to detect in this population [10,11,12]. Refugees were often exposed to traumatic experiences such as violence and loss of relatives [13]. During flight and in refugee camps, the risk factors accumulate and are reinforced by constant stress such as malnutrition and sleep deprivation [14]. Over the post-migration phase, refugees are burdened with additional challenges, including loss of social status, discrimination, language barriers, and a foreign culture [15]. Especially frequent is the occurrence of trauma sequelae such as post-traumatic stress disorders (PTSD), and depressions or anxiety disorders [10]. Accurate data on the prevalence of these vary widely, including rates of 5% for post-traumatic stress disorders up to 40% with evidence for psychiatric comorbidity [16,17].

At the same time, preventive and early detection examinations of asylum seekers and their children are less frequently used in Germany, while multifaceted barriers to access to healthcare are described [18].

Asylum seekers and those with a permit of residence, according to §§ 23 to 25 of the Residence Act, receive benefits according to the Asylum Seekers’ Benefits Act (*“Asylbewerberleistungsgesetz”*, AsylbLG) [19]. Their access to healthcare is restricted as compared to the majority population. Healthcare benefits for asylum seekers are based on §§ 4 and 6, which state that there is only acute and emergency care, treatment of pain, pregnancy- and birth care plus “necessary preventive measure” to grant [7]. Asylum seekers have to visit their social security office in order to obtain a healthcare-voucher which can be used to visit a doctor. Social security offices have been criticized for a lack of sufficient medical competence to decide about the need for medical treatment of a highly vulnerable group [20].

While an increase of visits to the doctor would lead to higher expenditures in the short run, it can also be viewed as one preventive part of health-related behavior regarding this population [21]. For this reason, it could lead to a decrease of expenditures in the long run, because chronification of diseases might be avoided.

At the end of 15 months, asylum seekers are granted benefits according to the twelfth Social Security Code. The § 2 AsylbLG was changed in 2014, so that asylum seekers now have to wait 15 months instead of 48 months in order to receive an Electronic Health Insurance Card (EHIC) which allows them to visit a doctor without being forced to show a healthcare-voucher [19].

Since 2015, each federal state of Germany can decide whether to hand out the EHIC to asylum seekers, right from the beginning of their stay. Therefore, they have to reach a framework agreement in negotiations with the states’ associations of health insurers [22].

By the end of 2017, except for Bavaria and Saxony, the German federal states have either decided for the introduction of the EHIC for asylum seekers or are currently in consultations. In North Rhine-Westphalia the municipalities are free to decide whether to introduce the EHIC for asylum seekers or not. Initially 16 municipalities including Bochum have introduced it by January 2016. The close-by cities Herne and Datteln still have not established it. This enables the comparison used in the study at hand.

North Rhine-Westphalia has to pay the health insurers a fixed rate of 8% a month per asylum seeker for the new incurred administration costs with 10 Euro being the minimum fee in any case. In return the health insurer has to inform the asylum seekers about the usage of the EHIC. Supporters argue that administration costs will decrease due to the disappearance of the obligation to decide about applications for healthcare-vouchers by asylum seekers at social security offices [23].

The number of benefits is still guided by §§ 4 and 6 AsylbLG. The criterion of deferability is not examined anymore, so that the treatment is now primarily indicated by the treating physicians, rather than employees of the social office. Instead, preventive cures, dentures, domestic aid, artificial insemination and sterilization, chronic disease management programs, selective tariffs as well as benefits abroad are explicitly excluded [24].

Critics argue that there is another incentive for migrating to Germany [25]. Further, an unregulated increase of healthcare costs is expected as a consequence of the fact that there is no incentive for the health insurers to reject benefits due to their own financial interest. Otherwise there is an incentive for healthcare providers to bill additional benefits when they already reached their Volume of Standard Benefits (*Regelleistungsvolumen*). Thus, the regular treatment of asylum seekers is not liable to budget restrictions [23].

Apart from that, the benefits are billed according to Uniform Assessment Standard (*Einheitlicher Bewertungsmaßstab*) instead of Medical Fee Schedule (*Gebührenordnung für Ärzte*). This means that the treatment of asylum seekers is controlled by Statutory Health Insurance and not viewed as a quasi-private service any longer [20].

As it is unknown in which direction an increase of the frequency of visits to the doctor by asylum seekers given the EHIC compared to a system of healthcare-vouchers affects overall healthcare costs, the study at hand aims to examine whether the mentioned increase is to be expected at all.

## 2. Study-Design & Methods

Due to a lack of available public data at this stage, asylum seekers themselves were asked in order to examine whether there is a difference in terms of ambulant visits to the doctor between asylum seekers with and without regular access to the German healthcare system. Null hypothesis states that there is no difference.

The study at hand used an ex-post-facto design via surveying 260 asylum seekers in the three municipalities Bochum, Datteln and Herne in North Rhine Westphalia, Germany. These were chosen due to Bochum being one of the municipalities which have introduced the EHIC for asylum seekers as soon as possible. Existing cooperation with Datteln and Herne simplified the access to the field in close-by municipalities. Ex-ante, it had been calculated that a minimum sample size of 210 cases would be sufficient to detect a medium effect size (d = 0.5), given a two-sided rejection region with a significance level of α = 0.05 and a statistical power of 95% if the two analyzed groups (with and without EHIC) are equally large [26]. The interviews were conducted in October 2016 and October 2017 using a standardized questionnaire in German, English, French, Arabic and Farsi with the support of interpreters. The survey was conducted on the respondents’ voluntary basis. All participants provided informed consent. They were approached by social workers, students or interpreters under supervision of the researchers. The questionnaire was processed independently and anonymously. In individual cases, comprehension questions were answered. At the time of the survey, the respondents lived in fourteen different community camps or urban facilities different from the reception centers of North Rhine-Westphalia.

Here, the count of visits to the doctor was inquired. Alongside the length of the stay in Germany up to the point of the survey, the period in which the respondents had been in possession of the EHIC was also asked for. Thus, the period prevalence can be determined for different doctor contacts, so that the monthly consultation rate of ambulant physicians (CR) acts as the main outcome. A total of 93 asylum seekers, who had used both healthcare-vouchers and the EHIC in order to see a physician, were treated as different cases who were in each system for a certain amount of time. Consequently, they have been taken into account in both groups. The CR was compared between the groups “with EHIC” and “without EHIC” by using a two-sided *t*-test with Welch-approximation due to the possibility of different variances [27]. It was assumed that the difference in population follows a t-distribution. Intraindividual differences were tested for within the group of 93 asylum seekers who had used both healthcare-vouchers and the EHIC over time. If only the count of healthcare-vouchers was remembered, the average count of visits to the doctor per healthcare-voucher was inputted.

Various other variables may represent a confounder in the comprehension of the physician contacts. So therefore, the presence of chronic diseases—whereby the most common chronic diseases are covered [28]—in the community, the months in Germany at the time of the survey, German language skills, age, sex, education and currently being on medication along with the date of the survey, country of origin and visits to the hospital or volunteering doctors were all factors especially considered and included as potential disturbers. In addition, the respondents rated their language skills by themselves using a scale from one (“very bad”) to five (“very good”). Education was measured as an ordinally scaled variable, ranging from one for no educational degree to five for a university degree. The date of the survey was also taken into account.

Those of the variables that show a Pearson correlation coefficient greater than 0.1 either with the count of visits to the doctor or with the ownership of the EHIC were controlled for using a linear OLS-regression model.

Goodness of fit was assessed by the adjusted coefficient of determination. Multicollinearity was tested for using variation inflation factors (VIF) assuming that those with values smaller than four were without problems [29]. Residual analysis was done by testing the assumptions of normal distribution and homoscedasticity using Shapiro–Wilk- and Breusch–Pagan-test. It was assumed that the residuals follow a normal distribution with constant variance [30].

Ex-ante, three observations were omitted completely for several values being either unrealistic or inconsistent. Item nonresponse was handled by mean imputation. Asylum seekers who used both healthcare-vouchers and the EHIC in order to see a physician were treated in the calculation of the period prevalence as different cases who were in each system for a certain amount of time.

## 3. Results

In total, 260 asylum seekers were interviewed in this study. A total of 137 of these persons were in possession of the EHIC. A total of 216 of the respondents were at least temporarily provided with healthcare-vouchers. Thus, 93 asylum seekers had been able to collect experiences with healthcare-vouchers as well as with the EHIC, because during their stay they first had been treated with healthcare-vouchers and after some time they received the EHIC.

The observation period of the group “with EHIC” thus consists of 2602.86 months. In contrast, the group without EHIC” shows an observation period of 2453.76 months.

The respondents were 31.12 years old on average with a range of 18 to 66 years if asylum seekers who had used both healthcare-vouchers and the EHIC are treated as different cases (pre and post EHIC). At 80%, the majority of them was male. With a total of 54%, Syria, Iraq and Afghanistan were the most frequently mentioned countries of origin. A total of 17% said they received a college degree, 5% vocational training and 25% the general qualification for university entrance. A total of 37% had not graduated from school. Further characteristics can be found in Table 1.

**Figure 1:**
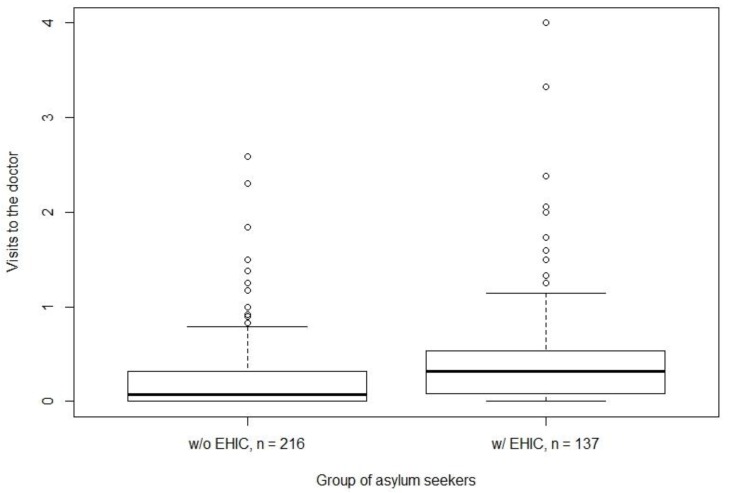
Consultation rate of ambulant physicians per month among a sample of asylum seekers in Germany stratified for ownership of the Electronic Health Insurance Card (EHIC) (with EHIC: *n* = 137, without EHIC: *n* = 216).

The mean CR was 0.32 ± 0.49 doctor contacts per month over all groups. As seen in Figure 1 owners of the EHIC showed a mean of 0.49 ± 0.65, while the mean of those who had to ask for healthcare-vouchers was 0.21 ± 0.39. The t-distributed mean difference of 0.28 is significant at *p* < 0.01. The 95% confidence interval includes mean differences between 0.14 and 0.41. The Pearson correlation coefficient was 0.25.

Within the group of asylum seekers who had used both the EHIC and healthcare-vouchers (*n* = 93), it was found that after receiving the EHIC, there was also a significant intra-individual increase of the CR (∆ = 0.27, *p* < 0.01).

Several chronic diseases, age, sex as well as the respondent’s level of education and country of origin as well as contacts to hospital and volunteering physicians showed a correlation smaller than 0.1 with the count of visits to the doctor and the ownership of the EHIC. They were omitted in the linear regression model.

A total of 71.95% were asked in Bochum, 23.23% in Datteln and 4.82% in Herne. The highest correlation (r = 0.29) was found between Bochum and the EHIC. At the same time, the CR was not affected by the municipality following correlations each smaller than 0.1.

Being on medication was positively associated with the CR (r = 0.25), whereas it was not associated with the ownership of the EHIC. At the time of the interview, 23.23% of the respondents were on medication, 73.94% were not; 0.04% did not answer the question. A total of 4.53% suffered from heart or other cardiovascular diseases. Cardiovascular diseases were correlated with the CR (r = 0.21), but not with the EHIC. The outcome´s correlation with cardiovascular diseases was outperformed by its correlation with psychiatric disorders (r = 0.24), from which 17% were suffering. A weaker association was discovered between the CR and joint diseases, from which 6.52% were suffering (r = 0.12). 

Months being in Germany up to the point of survey correlated with the ownership of the EHIC (r = 0.35), but not with the CR. German language skills were slightly correlated with the ownership of the EHIC (r = 0.11) and were thus entered into the regression model. The date of the survey was slightly correlated with the count of visits to the doctor (r = 0.12). A total of 39.09% of the respondents were asked in October 2016, and 60.91% in October 2017. 

A linear regression of the CR, the results of which can be found in Table 2, was done on the aforementioned predictors with correlations greater than 0.1. A proportion of 0.21 of the variance of the CR can be explained by the variance of the predicting variables. The adjusted R-Squared equals 0.19. The impact of the EHIC, psychiatric disorders and belonging to the municipality of Herne was positive and highly significant (*p* < 0.01). Ownership of the EHIC directly increased the CR by approximately 0.31 doctor contacts per month. The value of this regression coefficient exceeded the mean difference, which suggests that there is a mild suppression of the relationship of interest by the controlled for confounders. The positive effects of medication and cardiovascular diseases as well as the negative effect of being asked in 2017 were still significant on the 5% level.

The VIF ranged from 1.22 to 1.78, indicating that there was no severe multicollinearity. The Shapiro–Wilk-test signals that null hypothesis of normal distribution of residuals is to be rejected (*p* < 0.01). Moreover, the Breusch–Pagan-test suggests that there is heteroscedasticity (*p* < 0.01). Eliminating 25 cases with values of Cook greater than 4/n did not lead to the Breusch–Pagan null hypothesis being tenable [29]. These limitations of the statistical analysis must be taken into consideration regarding the interpretation of the results.

All in all, the availability of the EHIC presented itself as an independent predictor of the use of outpatient healthcare, because the CR was significantly lower in the group of patients “without EHIC” than in the group “with EHIC”.

## 4. Discussion

Those asylum seekers in Germany who were in possession of the EHIC due to living in Bochum or after 15 months of residence, visited their ambulant doctors significantly more often, as compared to the rest of the asylum seekers who had to ask the social security office for healthcare-vouchers.

Controlling for third variables indicates that the impact of the EHIC exists independent of the presence of chronic diseases, the municipality, months being in Germany up to the point of the survey, German language skills, age, sex, level of education, currently being on medication, the date of the survey, country of origin and visits to the hospital or voluntary doctors.

The study at hand is to be credited for its confirmation of the hypothesis that the introduction of the EHIC for asylum seekers nearly from the beginning of their residence does increase their monthly CR of ambulant physicians.

Nevertheless, the resulted sample of EHIC owning asylum seekers matches the Germans’ CR on average when the structure of age and sex is taken into consideration. The average German sees his/her ambulant doctors 9.2 times per year. Women visit their doctors 1.5 times per year more often on average than men. The relevant age group from 30 up to and including 39 shows a frequency of 7.7. If only men in this age group are viewed, their value is 5.7 which is equivalent to 0.48 visits per month, which does not significantly differ from the asylum seekers with EHIC [31].

Therefore, the results for unrestricted access are in line with previous research regarding the majority population, indicating that there is no significant difference between asylum seekers and the age-corrected autochthonous population in Germany in terms of visits to the doctor. In spite of that, it could be shown that the non-possession of the EHIC leads to a significantly lower use of the healthcare system among asylum seekers. Consequently, healthcare-vouchers can be viewed as a major obstacle in the medical care of this group.

Simultaneously, data on doctoral contacts by asylum seekers in Germany is scarce. Sönmez et al. interviewed 660 women in their “study on female refugees” in five German federal states. Only 16% of these women indicated that they have access to general medical care [32]. This can be interpreted as further evidence that there are systematic barriers to healthcare for asylum seekers in Germany.

Although the distribution of asylum seekers to the federal states, and further to the municipalities, takes place largely independent of person-specific characteristics, it has to be recognized that the results of the present study are limited locally and quantitatively. The clustered sample of the given number of cases, does not allow for drawing conclusions about the whole population of asylum seekers in Germany. However, an obvious trend can nonetheless be seen.

The central model explains a small amount of the overall variation of the outcome, while residuals do not scatter randomly. This could be due to decisive variables that were missing in the survey. In addition, there could have been interference effects, such as selection bias, e.g., absence due to parallel language courses, or that the mother tongue of the interviewed could not be covered by interpreters, or due to memory gaps.

What the survey does not provide is an educated guess about the actual costs of the increase of visits to the doctor. Although the EHIC for asylum seekers seems to remove barriers, it has to be detected in future research, whether the increase translates into higher overall costs. It can be supposed that, while healthcare costs will be increased in the short run, they will decrease in the long run due to the prevention and early treatment of possibly chronic diseases.

Fewer visits to the doctor for prevention or in the presence of mild to moderate symptoms may result in adverse health consequences for those affected, that would not only burden the people, but could also lead to significantly higher follow-up costs. Specifically, regarding the existing difficulties in the treatment of mental illnesses, such as PTSD, the occurrence of comorbidities (e.g., depression and addiction disorders) as a result of complications has to be expected due to a lack of adequate early treatment [33]. Their psychosocial effects can also lead to negative consequences for the social environment and society as a whole, i.e., in terms of integration by means of alienating a social group. In order to avoid medium- to long-term health consequences for asylum seekers as well as possible additional expenditures and adverse societal effects, it is worthwhile to provide an unrestricted access to healthcare.

This claim can be reinforced by the fact that, since the introduction of the EHIC for asylum seekers in Hamburg in 2012, the benefits remained constant while the administration costs could be decreased [34]. Moreover Bozorgmehr/Razum found out, by analyzing data from German Federal Statistical Office from 1994 to 2013, that restricting healthcare access of asylum seekers has generated additional costs in the average amount of 375.80 Euro per capita that lead to additional costs of 1.56 billion Euro for the whole period of time [35].

## 5. Conclusions

In this study, it could be shown that asylum seekers in possession of the EHIC are significantly more likely to seek ambulant medical care than those receiving healthcare-vouchers. Their CR, however, does not significantly differ from the age-corrected CR of the autochthonous population, as mentioned in the discussion section. Taking into account relevant covariables, the possession of the EHIC can be viewed as an independent influencing factor on the asylum seekers’ use of medical care. These results suggest that having to ask for healthcare-vouchers at the social security office could be a relevant barrier for asylum seekers. This could result in a lack of necessary treatment or precautionary measures, possibly resulting in complications or chronification. On the other hand, the ownership of the EHIC does not seem to lead to a more frequent use of medical services by the asylum seekers interviewed in the context of the study at hand if their count of visits to the doctor is compared to data of the German age-corrected total population. Further research from a health economical perspective—also with greater regional coverage—as well as the development of practice-oriented care approaches in order to reduce access barriers is therefore desirable.

## Figures and Tables

**Table 1:** Description of the main characteristics of the analyzed sample (*n* = 353; rounded percentages).

Characteristic	Percentage
Age in years (mean ± SD)	31.12 ± 9.49
Sex	
Male	80%
Country of origin	
Syria	30%
Iraq	14%
Afghanistan	10%
Iran	7%
Albania	1%
Eritrea	5%
Pakistan	2%
Serbia	1%
Other	30%
Family status	
Single	56%
Married	39%
Divorced	3%
Widowed	2%
Education	
College degree	17%
General qualification for university entrance	25%
Vocational training	5%
Other	16%
No educational degree	37%
Children	
None	60%
One child	9%
Two children	12%
More than two children	19%
Municipality	
Bochum	72%
Datteln	23%
Herne	5%
Point of the Survey	
2016	39%
2017	61%
Months in Germany (mean ± SD)	14.32 ± 10.54
German language skills	
1—very bad	18%
2—bad	23%
3—average	33%
4—good	21%
5—very good	4%
Currently on medication	
Yes	23%
Missing information	4%
Chronic Disease	
Yes	60%
Missing information	1%
Diseases	
Heart disease	5%
Psychiatric disorder	17%
Joint disease	7%
Diabetes	4%
Back pain	12%
Cancer	1%
Thyroid disease	3%
Other	20%
Electronic Health Insurance Card (EHIC)	
Yes	57%
Consultation rate of ambulant physicians (CR; visits to the doctor per month) (mean + SD)	0.32 ± 0.49
Contacts to further physicians	
Hospital	35%
Volunteers (e.g., in camp)	9%

**Table 2:** Factors associated with the consultation rate of ambulant physicians per month among a sample of asylum seekers in Germany (with EHIC: *n* = 137, without EHIC: *n* = 216).

Characteristic	Coefficient (Standard Error)	*P*-Value
Health system related variables		
Electronic Health Insurance Card (yes vs. no)	0.31 (0.06)	0.00
Medication (yes vs. no)	0.14 (0.06)	0.02
Language skills		
German (per increase on a scale from 1 to 5)	0.02 (0.02)	0.50
Diseases		
Heart disease (yes vs. no)	0.25 (0.13)	0.05
Psychiatric disorders (yes vs. no)	0.30 (0.07)	0.00
Joint disease (yes vs. no)	−0.03 (0.11)	0.81
Municipality		
Datteln (yes vs. no, compared to Bochum)	0.10 (0.07)	0.18
Herne (yes vs. no, compared to Bochum)	0.45 (0.12)	0.00
Time variables		
Point of the survey (2017, compared to 2016)	−0.16 (0.06)	0.01
Months in Germany (per additional month)	0.00 (0.00)	0.25

## Abbreviations

The following abbreviations are used in this manuscript:

EHIC	Electronic Health Insurance Card
CR	Consultation Rate of Ambulant Physicians
VIF	Variation Inflation Factors
PTSD	Post-Traumatic Stress Disorder
